# Supplementary material for: Multi‐dataset identification of innovative feature genes and molecular mechanisms in keratoconus
Source: J Cell Mol Med. 2024 Sep 19;28(18):e70079. doi: 10.1111/jcmm.70079 (PMC11412914; doi:10.1111/jcmm.70079)
Supplement: Supplementary file 2 — Material S2. [file JCMM-28-e70079-s002.zip › 7.GSEA analysis/4.MYRF/KEGG_INTESTINAL_IMMUNE_NETWORK_FOR_IGA_PRODUCTION.html]

Details for gene set KEGG\_INTESTINAL\_IMMUNE\_NETWORK\_FOR\_IGA\_PRODUCTION[GSEA]

|  || Dataset | MYRF.group.cls#h\_versus\_l |
| Phenotype | group.cls#h\_versus\_l |
| Upregulated in class | l |
| GeneSet | KEGG\_INTESTINAL\_IMMUNE\_NETWORK\_FOR\_IGA\_PRODUCTION |
| Enrichment Score (ES) | -0.58822954 |
| Normalized Enrichment Score (NES) | -1.5871176 |
| Nominal p-value | 0.006024096 |
| FDR q-value | 0.21933451 |
| FWER p-Value | 0.633 |
Table: GSEA Results Summary

  

Fig 1: Enrichment plot: KEGG\_INTESTINAL\_IMMUNE\_NETWORK\_FOR\_IGA\_PRODUCTION      
 Profile of the Running ES Score & Positions of GeneSet Members on the Rank Ordered List

  

| SYMBOL | TITLE | RANK IN GENE LIST | RANK METRIC SCORE | RUNNING ES | CORE ENRICHMENT || 1 | CD86 | na | 355 | 0.626 | 0.0408 | No |
| 2 | ITGA4 | na | 1618 | 0.389 | 0.0361 | No |
| 3 | TNFRSF13C | na | 5257 | 0.215 | -0.0513 | No |
| 4 | TNFSF13B | na | 6312 | 0.183 | -0.0668 | No |
| 5 | ICOSLG | na | 7776 | 0.144 | -0.0972 | No |
| 6 | HLA-DOA | na | 8167 | 0.134 | -0.0975 | No |
| 7 | ITGB7 | na | 9321 | 0.109 | -0.1219 | No |
| 8 | TNFRSF13B | na | 10434 | 0.088 | -0.1468 | No |
| 9 | ICOS | na | 11751 | 0.067 | -0.1793 | No |
| 10 | CCR10 | na | 12225 | 0.060 | -0.1880 | No |
| 11 | CD28 | na | 12283 | 0.059 | -0.1848 | No |
| 12 | AICDA | na | 16059 | 0.017 | -0.2923 | No |
| 13 | HLA-DRB5 | na | 20183 | -0.024 | -0.4092 | No |
| 14 | TNFRSF17 | na | 21464 | -0.039 | -0.4429 | No |
| 15 | IL15RA | na | 21497 | -0.040 | -0.4406 | No |
| 16 | CCL27 | na | 22822 | -0.057 | -0.4741 | No |
| 17 | CXCL12 | na | 22943 | -0.059 | -0.4728 | No |
| 18 | HLA-DRB3 | na | 23156 | -0.062 | -0.4739 | No |
| 19 | LTBR | na | 23190 | -0.063 | -0.4697 | No |
| 20 | HLA-DQB1 | na | 24038 | -0.076 | -0.4879 | No |
| 21 | HLA-DRB1 | na | 24046 | -0.077 | -0.4819 | No |
| 22 | HLA-DOB | na | 24105 | -0.078 | -0.4772 | No |
| 23 | TNFSF13 | na | 24888 | -0.091 | -0.4923 | No |
| 24 | IL5 | na | 26211 | -0.117 | -0.5209 | No |
| 25 | CD80 | na | 26452 | -0.122 | -0.5178 | No |
| 26 | CCL28 | na | 26883 | -0.132 | -0.5194 | No |
| 27 | HLA-DMA | na | 27498 | -0.148 | -0.5251 | No |
| 28 | CCR9 | na | 28216 | -0.167 | -0.5322 | No |
| 29 | IL10 | na | 28824 | -0.186 | -0.5346 | No |
| 30 | HLA-DRB4 | na | 30247 | -0.232 | -0.5566 | No |
| 31 | CD40LG | na | 30708 | -0.251 | -0.5495 | No |
| 32 | HLA-DPA1 | na | 32053 | -0.320 | -0.5621 | Yes |
| 33 | CD40 | na | 32572 | -0.356 | -0.5481 | Yes |
| 34 | PIGR | na | 32602 | -0.357 | -0.5198 | Yes |
| 35 | MAP3K14 | na | 32615 | -0.359 | -0.4909 | Yes |
| 36 | TGFB1 | na | 32868 | -0.377 | -0.4674 | Yes |
| 37 | HLA-DRA | na | 33031 | -0.390 | -0.4403 | Yes |
| 38 | IL4 | na | 33045 | -0.391 | -0.4088 | Yes |
| 39 | IL15 | na | 33083 | -0.394 | -0.3777 | Yes |
| 40 | HLA-DMB | na | 33256 | -0.414 | -0.3489 | Yes |
| 41 | IL6 | na | 33353 | -0.425 | -0.3170 | Yes |
| 42 | IL2 | na | 33402 | -0.431 | -0.2833 | Yes |
| 43 | MADCAM1 | na | 33498 | -0.443 | -0.2500 | Yes |
| 44 | HLA-DQA2 | na | 34217 | -0.576 | -0.2237 | Yes |
| 45 | HLA-DPB1 | na | 34331 | -0.609 | -0.1773 | Yes |
| 46 | CXCR4 | na | 34584 | -0.727 | -0.1253 | Yes |
| 47 | HLA-DQA1 | na | 34602 | -0.745 | -0.0651 | Yes |
| 48 | CCL25 | na | 34662 | -0.842 | 0.0019 | Yes |
Table: GSEA details [plain text format]

  

Fig 2: KEGG\_INTESTINAL\_IMMUNE\_NETWORK\_FOR\_IGA\_PRODUCTION      
 Blue-Pink O' Gram in the Space of the Analyzed GeneSet

  

Fig 3: KEGG\_INTESTINAL\_IMMUNE\_NETWORK\_FOR\_IGA\_PRODUCTION: Random ES distribution      
 Gene set null distribution of ES for **KEGG\_INTESTINAL\_IMMUNE\_NETWORK\_FOR\_IGA\_PRODUCTION**
